# High-plex protein imaging across thick tissue by synchronized amplification

**DOI:** 10.64898/2026.10.12.757405

**Published:** 2026-10-14

**Authors:** Kuanwei Sheng, Hanquan Su, Gokul Gowri, Maxwell Furman, Matthew Serrata, Lili Du, Charlotte George, Michel Nofal, Ryan McMillan, Kaibo Ma, Xiaokang Lun, Hiroshi Sasaki, Jiyoun Jeong, German Pihan, Peng Yin

## Abstract

High-plex protein imaging is essential for decoding complex tissue organization, yet it remains largely confined to thin sections. Current approaches in millimeter-thick samples suffer from poor signal penetration and depth-dependent, uneven signal amplification. Even with specialized instrumentation to facilitate probe diffusion, iterative millimeter-thick tissue workflows can take more than four days per staining round, and reported eleven-marker experiments extend beyond a month. We introduce Amplification by Cyclic Extension (ACE), a DNA-barcoded, synchronized *in situ* amplification strategy that uses thermally controlled cyclic extension to coordinate DNA concatemer growth across tissue depths. By synchronously activating and coordinating reaction progression throughout the sample, ACE enables controlled, uniform signal amplification across tissue depths. Using ACE, we demonstrate 63-plex protein and RNA imaging in thin sections, 22-plex protein imaging in 200 µm mouse brain sections, and 16-plex protein imaging in 1 mm-thick brain tissue encompassing over three million cells. For millimeter-thick tissues, ACE reduces per-round target-switching time from multiple days to less than eight hours, shifting volumetric high-plex protein imaging from months to days. Together, these results establish ACE as a synchronized amplification strategy that enables scalable high-plex protein imaging in thick tissues.

